# The role of social class in academic university contexts: exploring students' academic self-concept, educational expectations, and achievement goals

**DOI:** 10.3389/fpsyg.2025.1542359

**Published:** 2025-07-10

**Authors:** Mara Marini, Chiara Parisse, Stefano Pagliaro, Ilaria Giovannelli, Davide Pietroni, Stefano Livi

**Affiliations:** ^1^Department of Neuroscience, Imaging and Clinical Sciences, University of Chieti-Pescara, Chieti, Italy; ^2^Department of Social and Developmental Psychology, Sapienza University of Rome, Rome, Italy; ^3^Department of Psychology, University of Chieti-Pescara, Chieti, Italy

**Keywords:** social class, first-generation students, subjective social status, academic self-concept, educational expectations, achievement goals

## Abstract

**Introduction:**

Despite international progress, socioeconomic inequalities continue to significantly impact young people's opportunities to realize their full potential and actively engage in society. Research has demonstrated that socioeconomic factors are critical risk factors for undergraduate students' adaptation and academic success. However, the psychological processes underlying this phenomenon remain inadequately understood.

**Methods:**

To address this gap, we conducted two studies involving university students. Study 1 explored the relationship between socioeconomic status (both objective [parents' educational attainment] and subjective [subjective social status] indicators), students' academic self-concept, and educational expectations. In Study 2, we investigated the moderating role of achievement goals in the relationship between socioeconomic status and academic self-concept.

**Results:**

The findings indicated that socioeconomic barriers were negatively related to students' educational expectations through academic self-concept. Additionally, low performance-avoidance goals were found to enhance first-generation students' academic self-concept.

**Discussion:**

These results underscore the importance of supporting students from low socioeconomic backgrounds, thereby improving their self-perceptions in academic settings and assisting them to achieve their educational goals.

## 1 Introduction

Despite the growing global commitment to addressing educational and career inequalities (Agenda, 2030), structural factors—that is, individual characteristics that cannot be changed through personal effort (OECD, 2024a)—continue to play a crucial role in shaping young people's life experiences (OECD, 2024a,b). Even when other structural variables (e.g., gender or migration background) are accounted for, socioeconomic status (SES) remains a significant barrier to success in the educational and career paths of young people (Eriksson et al., 2021; OECD, 2023), including those who reach higher education (OECD, 2024a).

Notwithstanding the increasing participation of young people in higher education, including non-traditional students (Kim, 2007), such as first-generation students (i.e., those who are the first in their family to attend university) and students from low socioeconomic backgrounds (Marginson, 2016; OECD, 2024a), numerous studies, literature reviews, and meta-analyses have shown that these expanded-access opportunities have not reduced the socioeconomic disparities in students' academic and career outcomes. Students from low-income families and first-generation students tend to perform academically worse than their more advantaged peers, exhibit higher dropout rates, and achieve lower learning outcomes (e.g., Cifuentes Gomez and Santelices, 2024; Liu et al., 2022; López et al., 2023; OECD, 2019, 2023, 2024a; Van Ewijk and Sleegers, 2010). However, the mechanisms through which family SES influences students' educational and professional pathways remain poorly understood (Korous et al., 2022). This gap may be due to the limited attention given to the role of motivational variables in the relationship between SES and academic outcomes, especially in the context of higher education. In this regard, recent psychological literature has highlighted the importance of considering the influence of social and cultural factors, including SES, on human motivation (Eccles and Wigfield, 2020), particularly concerning perceived academic competence (Fang et al., 2018; Wu et al., 2021) and achievement goals (Elliot and Hulleman, 2017; Urdan and Kaplan, 2020). With the present research, we aimed to contribute to this growing body of literature by exploring the psychological processes that connect SES to undergraduate students' adaptation.

## 2 Theoretical background

In educational research, family SES is mainly assessed through parents' educational attainment (Diemer et al., 2013; OECD, 2023, 2024a,b). However, social class includes both objective (also referred to as SES, typically encompassing educational level, income, and occupational prestige) and subjective (e.g., subjective social status) indicators which reflect individuals' socioeconomic and cultural positions within their social context, as well as the social, economic, and cultural resources available to them (Diemer et al., 2013; Tan et al., 2020). In particular, it has been shown that subjective social status predicts wellbeing across various contexts more accurately than objective measures of social class (Tan et al., 2020). While objective and subjective social status indicators are moderately correlated (Tan et al., 2020), research has demonstrated that subjective social status is a significant explanatory factor for various aspects of students' psychological experiences, including confidence in their ability to achieve educational and career goals (Flores et al., 2017; Garriott et al., 2017; Marini et al., 2023).

The role of SES in students' experiences remains inadequately explored in educational psychology research. This is partly due to the common practice of treating socioeconomic indicators, such as parental educational attainment, as control variables in research models and the limited attention paid to the subjective indicators of social class (Diemer et al., 2013). Moreover, research has predominantly examined the associations between SES and academic achievements like grades or standardized test scores. This focus has led to an overlooking of the potential impact of SES on various psychological dimensions (Jury et al., 2017; Korous et al., 2022; Michael and Kyriakides, 2023). For these reasons, recent studies have increasingly emphasized the importance of considering motivation factors (e.g., achievement goals, academic self-concept) as critical elements in understanding the relationship between SES and academic achievement.

### 2.1 SES and perceived academic competencies

Although perceived competence has been understood differently within motivational theories, research has indicated that feeling competent in a specific area, such as academic contexts, is essential for individual wellbeing (Marsh et al., 2017). In the Marsh/Shavelson model (Marsh, 1990; Marsh et al., 1988), academic self-concept (ASC) is a component of students' overall self-concept, focusing on how students perceive their abilities and competencies in academic settings. A positive ASC is generally associated with higher academic performance, while a negative ASC may lead to lower achievement and academic disengagement (Marsh et al., 2017). Students who perceive themselves as academically competent (i.e., with a high ASC) tend to perform better than their less confident peers, which, in turn, further reinforces their sense of competence (Wu et al., 2021). Regarding its antecedents, ASC can be influenced by both learning-environment characteristics (e.g., classroom goal structures; Murayama and Elliot, 2009) and how students interpret their experiences within these environments (e.g., social-comparison processes; Marsh et al., 2017). Indeed, research findings indicate that ASC is not solely determined by individual performance (e.g., grades) but is also considerably influenced by the comparison of that performance with that of others (see “Big Fish Little Pond Effect”; Marsh and Seaton, 2015). In terms of outcomes, ASC is linked to a range of positive results, extending beyond grades—a factor that has been widely studied—to include various short- and long-term positive psychological perceptions and experiences (Marsh et al., 2023).

Regarding the relations between SES and ASC, some studies have shown that students from more privileged socioeconomic backgrounds have more favorable perceptions of their academic competencies, which, in turn, favor academic success in both middle and high school (Chevalère et al., 2023; King et al., 2024; Li et al., 2020; Marsh et al., 2023). These educational disparities have been interpreted through a wide range of models emerging from diverse theoretical frameworks and scientific disciplines. Moving beyond a deficit-based perspective—which attributes underachievement among students from low socioeconomic backgrounds to individual characteristics (e.g., low intelligence or lack of motivation)—contemporary theoretical paradigms increasingly underscore the role of academic environments in perpetuating educational socioeconomic inequalities (Goudeau et al., 2025). Across all levels of education, academic environments, which are shaped by meritocratic beliefs (e.g., Butera, 2006) and by the values and cultural norms of the dominant social classes (i.e., middle and upper classes) (e.g., Kraus and Stephens, 2012; Stephens et al., 2012), promote the idea that academic success mainly depends on individual effort and perseverance, regardless of broader structural factors. In this context, Butera (2006) and several other scholars interested in understanding the factors that promote or hinder equality in education (e.g., Darnon et al., 2018a,b; Goudeau et al., 2025) have described the concept of meritocracy in schools as a serious obstacle to the academic success of students from impoverished socioeconomic backgrounds. These scholars argue that the idea of giving all students the same opportunities and evaluating them using uniform criteria (e.g., grades) guarantees equal outcomes may undermine students' self-concepts. Students from less privileged backgrounds who lack access to the same resources as their more advantaged peers may struggle more with learning, which can lead to poor academic performance. Within a meritocratic system, these students may believe their underachievement is due to a lack of effort or ability; as a result, they may internalize their failure and develop a lower sense of competence (Chevalère et al., 2023; King et al., 2024; Li et al., 2020).

Despite these findings, the relationship between SES and ASC in academic university contexts remains underexplored, particularly concerning its implications for students' educational expectations. In fact, most studies on ASC have focused on its effects on academic achievement, with less attention paid to psychosocial variables, such as educational expectations. This gap in the literature is particularly important given that international surveys have shown that students from low-SES families are more likely to face challenges in achieving success regardless of their academic performance (OECD, 2024a).

### 2.2 SES and achievement goals

Achievement goals (AGs)—which represent the reasons that drive students to engage in academic activities—have a profound influence on students' educational experiences (Urdan and Kaplan, 2020). Achievement goal theory identifies four distinct categories of AGs: performance-approach (PAp) goals, mastery-approach (MAp) goals, performance-avoidance (PAv) goals, and mastery-avoidance (MAv) goals (Elliot, 1999; Elliot and McGregor, 2001; Pintrich, 2000; Senko, 2016). PAp and MAp goals are typically considered adaptive motivational orientations (Ames and Archer, 1988; Elliot, 1999; Elliot and Hulleman, 2017; Huang, 2012). Students with high levels of PAp goals are motivated to engage in educational contexts to demonstrate their abilities and competencies. Generally, these students believe competence cannot be developed through commitment and effort and hold fixed beliefs about intelligence and personality (Ames, 1992; Dweck and Leggett, 1988; Nicholls, 1984). In contrast, MAp goals are based on intrapersonal standards, fostering the perception of greater control over one's learning. These students are motivated to learn as much as possible and develop their skills to the best of their ability (Elliot and Hulleman, 2017). While earlier research classified PAp goals as maladaptive, emerging evidence suggests that both the approach and mastery components of AGs are positively associated with academic achievement (Huang, 2012). By contrast, the avoidance dimensions of AGs are generally considered maladaptive. When students engage in academic activities to avoid appearing less competent than their peers (PAv goals) or out of fear of not meeting their learning expectations (MAv goals), the quality of their learning and engagement tends to decline (Elliot, 1999; Elliot and Harackiewicz, 1996). PAv goals have been shown to reduce intrinsic motivation and self-perceived competence while simultaneously intensifying negative emotional states like school anxiety (Elliot and Church, 1997; Elliot et al., 1997; Mouratidis et al., 2013). There is less research on MAv goals, but since the beneficial effects of a mastery orientation are combined with avoidance dimensions, such goals are generally considered maladaptive (Elliot, 1999).

Recent studies have identified significant relationships between SES and AGs, often attributed to cognitive processes triggered by social memberships (e.g., Berger and Archer, 2015). In line with the socio-cognitive theory of social class (Kraus and Stephens, 2012; Stephens et al., 2012), students from higher socioeconomic backgrounds—who have multiple resources that make them more autonomous and less dependent on their environment—would interpret reality and experiences through a solipsistic socio-cognitive approach characterized by a strong orientation toward the self, personal needs, and individual interests. These socio-cognitive tendencies would enable these students to maximize the benefits of MAp goals, as they have the necessary resources to focus on self-actualization and intrinsic motivation in academic contexts. In contrast, students from lower socioeconomic backgrounds—who perceive less control over their environment due to their lower status within the social hierarchy—are more likely to develop other-oriented mindsets and contextualistic socio-cognitive tendencies (Kraus and Stephens, 2012; Stephens et al., 2012), as they depend more on others to achieve their goals than students from higher SES backgrounds. Additionally, the heightened attention to potential threats and obstacles to academic success would explain why low-SES students are more likely to evaluate their progress in comparison to that of their peers, thus making them more inclined to benefit from PAp goals (Berger and Archer, 2015, 2018).

From a different perspective, in academic environments, the common belief that success mainly depends on individual effort and perseverance can increase competition among students (Darnon et al., 2023), influencing the relationship between SES and AGs. In competitive environments, in fact, students from low socioeconomic backgrounds may face disadvantages in their academic performance. These students—who often have limited access to material, social, and economic resources—are usually raised with values of interdependence (Kraus and Stephens, 2012; Stephens et al., 2012), according to which building strong relationships and helping each other are essential to successfully cope with difficulties (see Goudeau et al., 2025, for details on how cultural differences can affect students' educational experiences). As a result, these students may not be fully prepared for academic environments where, unlike their home values, independence—a value typical of higher social classes—is strongly emphasized (Goudeau et al., 2025; Kraus and Stephens, 2012; Stephens et al., 2012). Therefore, AGs that rely on social comparison and foster a competitive mindset, such as performance goals (Ames, 1992; Ames and Archer, 1988), appear to offer limited benefits for the academic adaptation of students of low SES.

However, such theoretical assumptions have not always been confirmed in the educational literature. While Berger and Archer (2018) found that students of lower SES are less motivated than their higher-SES peers, this evidence does not emerge in academic university contexts. For example, Darnon et al. (2018a,b) observed no significant differences in AGs (PAp and MAp goals) based on social class (first-generation vs. continuing-generation students), suggesting that these two constructs are independent and may interact to predict achievement-related outcomes (also see Smeding et al., 2013). In particular, PAp goals were more beneficial for continuing-generation students, as they were better-suited to low-uncertainty situations, typical of the life experiences of high-SES students. Regarding PAv goals, Bruno et al. (2019) showed that in academic university contexts, the avoidance dimension of performance goals was negatively related to achievement outcomes, particularly for low-SES students (first-generation students). However, a recent meta-analysis on the relationship between economic status and avoidance motivation revealed minimal associations, with no significant findings from undergraduate student samples or studies measuring AGs (Gilbert et al., 2022).

### 2.3 Achievement goals, perceived academic competencies, and SES

The studies examined to date have underscored the pivotal role of motivational variables, including AGs and ASC, in shaping the developmental trajectories of young individuals, particularly in the presence of adverse conditions such as low SES.

Regarding the interplay between AGs and ASC, empirical evidence has indicated that AGs focus on social comparison (i.e., when students' motivation is oriented toward demonstrating their abilities [PAp goals] or avoiding the appearance of incompetence [PAv goals] relative to their peers) are linked to ASC (Niepel et al., 2014; Wirthwein and Steinmayr, 2021). Indeed, these motivational dimensions share a common emphasis on academic achievement (one's own and those of others) as a central driver of motivated behavior (Elliot et al., 2017). Furthermore, MAp goals have been found to exhibit a positive association with ASC (e.g., Niepel et al., 2014; Wouters et al., 2015).

Regarding the role of socioeconomic factors in motivated behavior, no studies have specifically addressed the relationship between AGs and ASC, taking into account students' SES. The majority of the existing research has concentrated on the interaction between SES and AGs in relation to academic achievement, often overlooking their potential impact on broader psychological constructs like ASC. Given the bidirectional relationship between academic achievement and ASC (Wu et al., 2021), it is reasonable to infer that these associations hold significance even when exploring the psychological correlates of academic performance (i.e., ASC). Specifically, King et al. (2024) found that MAp goals were linked to high academic performance across all students but were particularly beneficial for enhancing perceptions of academic competence among students of higher SES (measured through a composite index of household assets, parental employment status, and parental education level). In addition to their role in promoting academic adaptation, MAp goals seem to amplify the positive effects associated with belonging to affluent families. As suggested by Berger and Archer (2015), students of high SES are in the best position to benefit from MAp goals due to their access to a broader range of economic, social, and cultural resources, greater control over their environment, and a stronger motivation to reach their full potential (Kraus and Stephens, 2012). However, in academic university contexts, MAp goals seem to benefit low-SES students (first-generation students), particularly concerning their academic achievement (Darnon et al., 2018a,b). These results are consistent with numerous studies that applied achievement goal theory in educational contexts, indicating that MAp goals are essential for successful adaptation in school settings and students' wellbeing (Diaconu-Gherasim et al., 2024; Huang, 2016). Consequently, given the considerable variability in studies on this topic, which differ by educational level and the methods used to measure students' adaptation processes, further research is needed to clarify the relationships between SES, ASC, and AGs.

## 3 The present research

With this research, we aimed to investigate the role of social class in students' academic adaptation. Particularly, while educational research had primarily measured SES in terms of parents' educational attainment, our research also considered subjective social status (Diemer et al., 2013). Compared to objective SES indicators, this construct reflects the subjective perception of one's social, economic, and cultural resources (Diemer et al., 2013), providing a more accurate representation of personal social status. In addition, we focused on the psychological mechanisms involved in the relationship between SES and students' academic adaptation, taking into account ASC, educational expectations, and AGs. In fact, most research on this topic has primarily considered academic achievement as an indicator of students' academic success, neglecting the role of SES in broader students' psychological experiences (Michael and Kyriakides, 2023). In order to overcome these literature gaps and fulfill our goal, we conducted two studies.

In *Study 1* we investigated the association between students' social class and their educational expectations, considering the role of ASC in this relationship. In this regard, the literature has shown that students of lower SES tend to have lower ASC (Chevalère et al., 2023; King et al., 2024; Li et al., 2020; Marsh et al., 2023). However, this relationship remains relatively unexplored within academic university settings, especially regarding its implications for psychological dimensions, such as educational expectations.

Building on the results of Study 1, in *Study 2* we explored whether AGs could explain the association between students' social class and ASC. Some prior research had indicated that AGs are implicated in the relationship between social class and academic success, both in terms of academic achievement (Bruno et al., 2019; Darnon et al., 2018a,b; Smeding et al., 2013) and ASC (King et al., 2024). Investigating the role of AGs in the direct association between social class and academic adaptation is crucial for identifying potential risk and protective factors that may shape this relationship.

## 4 Study 1

In this study, we investigated the role of both objective SES (parents' educational attainment) and subjective SES (subjective social status) in relation to ASC and students' educational expectations. Specifically, we explored whether these social class indicators were associated with ASC and, through this, with students' expectations of successfully completing the current course of study. Based on the literature reviewed above, we hypothesized that lower social class (being a first-generation student and having a low subjective social status) would be negatively associated with ASC, which, in turn, would be positively associated with students' educational expectations. We also hypothesized an indirect association between students' social class and educational expectations *through* ASC.

Although no studies have compared the role of objective and subjective indicators of social class in students' adaptation with respect to the variables examined in this study, we hypothesized that the associations between these variables would be stronger when subjective indicators are considered. Indeed, compared to parents' educational attainment, subjective SES indicators more effectively capture the social, economic, and cultural resources available to students and their families. Therefore, beyond the influence of subjective social status, we hypothesized that the impact of students' generational status on the examined processes would be minimal.

### 4.1 Method

#### 4.1.1 Participants

An a priori power analysis indicated that with a critical alpha of *p* = 0.05 and an effect size between 0.25 and 0.30 in a structural equation model with two observed exogenous variables, one latent endogenous mediator and one latent endogenous dependent variable (estimated factor loading = 0.70), 250 participants were sufficient to achieve a minimum power of 0.80 (1,000 replications) (Wang and Rhemtulla, 2021).

A total of 263 Italian undergraduate psychology students (*M*_age_ = 20.63, *SD*_age_ = 2.09; 68.1% female, 39.7% male, and 0.4% non-binary; five participants did not report gender information; six participants did not report their age) participated in this study. Informed consent was obtained from all participants before starting the online survey. This study was approved by the university ethics committee and adhered to ethical standards for psychological research.

#### 4.1.2 Measures

Students' social class was measured with both objective and subjective indicators. Regarding objective indicators, and in line with the educational literature, students' generational status was assessed. Students reported the highest level of education attained by their parents (from elementary school to a doctoral degree). Participants with at least one parent with a bachelor's degree were classified as continuing-generation (CG) students (*N* = 130). Students whose parents did not have a bachelor's degree were classified as first-generation (FG) students (*N* = 132). One student did not report their parents' educational attainment. As a subjective indicator of social class, subjective social status was measured with an adaptation to the Italian context of the MacArthur Scale of Subjective Social Status (Adler et al., 2000). Students assessed their family's social status by responding to the following item on a scale from 1 (low subjective perceived social status) to 10 (high subjective perceived social status): “*Please consider this scale to describe your position in society. At the tenth step of the scale, you will find those in high social positions, with abundant financial resources, high levels of education, and prestigious professional positions. At the bottom of the scale (step 1), individuals occupy lower positions, with fewer economic resources, low levels of education, and less prestigious jobs or no employment. Choose the position on the scale that most accurately reflects your social position, taking into account your family of origin”*. The scale scores were reversed so that higher scores indicated lower subjective social status.

ASC was assessed with five items designed to measure perceptions of academic competence (Marsh, 1990), which were adapted to the university context (example item: “I obtain good grades in my university courses”; 8-point Likert scale; ω = 0.84). Confirmatory factor analysis revealed a good fit of the one-factor model to the data ($\chi_{\left(5\right)}^{2}$ = 2.107, *p* = 0.834, CFI = 1, TLI = 1, RMSEA = 0.00, SRMR = 0.01).

Students' expectations of successfully completing their course of study were measured with three items created specifically for this study (example item: “I am confident that I will successfully complete my course of study”), to which students responded on a 5-point Likert scale. Reliability was adequate (ω = 0.84).

### 4.2 Data analysis and results

**Figure 1:**
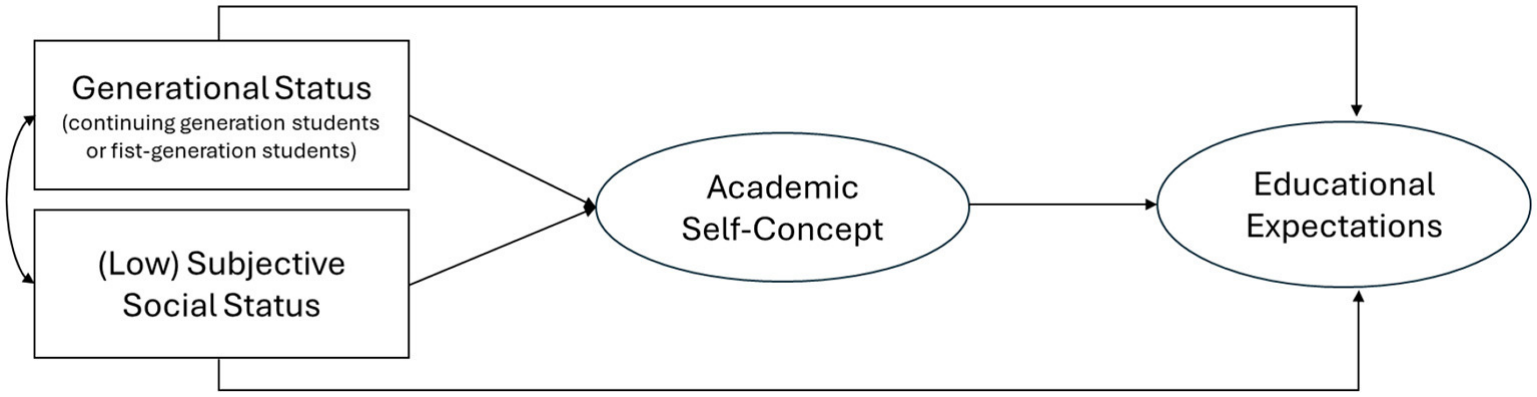
Conceptual model.

We conducted all analyses in R Core (R Core Team, 2024), using the lavaan package for path analysis (Rosseel, 2012) and the semTools package for estimating the indirect effects (Jorgensen et al., 2022). After performing descriptive statistics and preliminary analyses, we evaluated a structural equation modeling (maximum likelihood estimator) in which the social class indicators (generational status and subjective social status) were included as observed exogenous variables, while ASC (mediator) and educational expectations (dependent variable) were included as latent endogenous variables (see Figure 1). The model fit was evaluated using the following indices: TLI (Tucker-Lewis Index), CFI (Comparative Fit Index), RMSEA (Root Mean Square Error of Approximation), and SRMR (Standardized Root Mean Square Residual). TLI > 0.90, CFI > 0.95, and RMSEA and SRMR values ≤ 0.08 were considered indicators of a good fit (Hu and Bentler, 1999). The indirect associations between social class and students' educational success expectations via ASC were assessed with Monte Carlo 95% confidence intervals (CIs), with 50,000 draws.

Before performing our analyses, we inspected the bivariate correlations (see Table 1). Consistent with the literature (Tan et al., 2020), objective and subjective indicators of social class were positively correlated. FG students reported lower levels of subjective social status (*M* = 5.96, *SD* = 1.44, *N* = 132) compared to CG students (*M* = 6.95, *SD* = 1.18, *N* = 130) (Student's *t*-test = 6.124, *p* < 0.001). Generational status was not correlated with either ASC or educational expectations. Low subjective social status was negatively correlated with ASC and educational expectations. ASC and educational expectations were positively correlated. No gender differences emerged in ASC or educational expectations.

**Table 1:** Descriptive statistics and correlation analyses.

**Variables**	**M**	**SD**	**1**	**2**	**3**	**4**
1. GS	–	–	–			
2. L-SSS	4.55	1.40	0.355^***^	–		
3. ASC	5.69	1.24	.038	−0.201^**^	–	
4. EE	3.96	0.82	0.044	−0.157^*^	0.642^***^	–

* *p* < 0.05.

** *p* < 0.01.

*** *p* < 0.001.

GS, generational status (0 = Continuing-Generation; 1 = First-Generation); L-SSS, low subjective social status; ASC, academic self-concept; EE, educational expectations.

**Figure 2:**
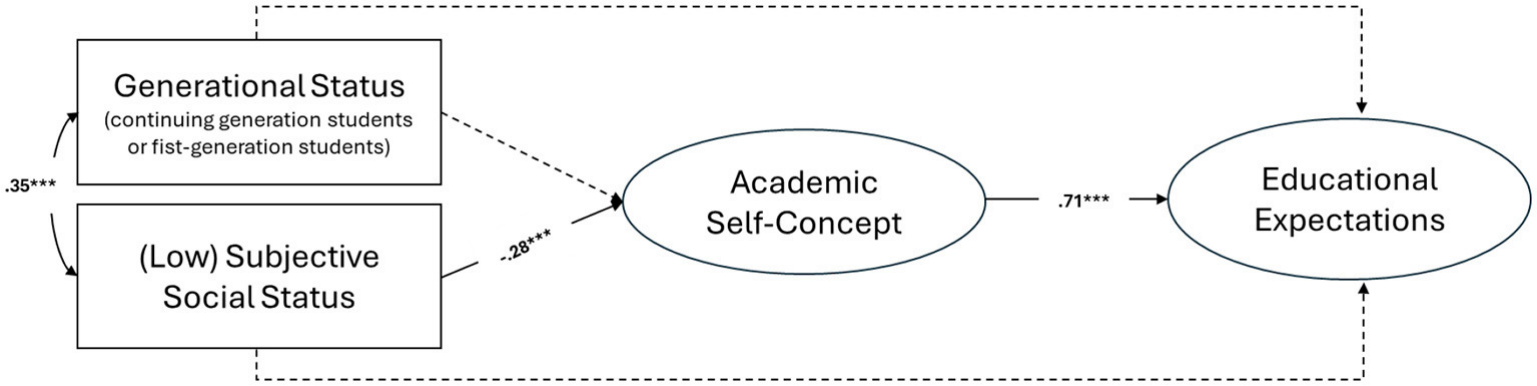
Structural equation model results. ***p < 0.001. Generational status (0 = Continuing-Generation; 1 = First-Generation). Dotted lines indicate non-significant associations.

The model in Figure 2 showed adequate fit indices ($\chi_{\left(31\right)}^{2}$ = 67.734, *p* < .001, CFI = 0.97, TLI = 0.95, RMSEA = 0.07, SRMR = 0.04).

The findings showed that generational status (0 = CG, 1 = FG) and low subjective social status were positively associated (β = 0.35, *p* < 0.001). A negative association emerged between low subjective social status and ASC (β = −0.28, *p* < 0.001). Generational status was not associated with ASC (β = 0.13, *p* = 0.053). However, it is interesting to note that this association, while marginally significant, was positive. This suggests that achieving higher education, despite limited social, economic, and cultural resources within a family, may contribute to positive self-perceptions regarding students' academic competencies. The results also showed a significant positive association between ASC and students' educational expectations (β = 0.71, *p* < 0.001). No significant associations were found between generational status (β = 0.03, *p* = 0.632) or low subjective social status (β = −0.04, *p* = 0.522) and students' educational expectations. The results indicated that only low subjective social status was negatively associated with students' educational expectations indirectly via ASC (β = −0.20, 95% CI: −0.29, −0.10). Generational status and students' educational expectations were not indirectly associated via ASC (β = 0.09, 95% CI: −0.01, 0.19). The model explained 6.7% of the variance in ASC and 51.8% of the variance in students' educational expectations.

### 4.3 Discussion

In this study, we explored psychological processes underlying the associations between social class and students' adaptation in academic university contexts. In contrast to prior research on this topic, the focus of this study was on ASC and educational expectations rather than academic achievement. Indeed, even when academic performance is equivalent, students from disadvantaged socioeconomic backgrounds tend to experience lower levels of academic and professional success compared to their peers from more privileged socioeconomic backgrounds (OECD, 2024a). Therefore, it is important to examine the psychological factors, particularly motivational variables, that may help explain these educational disparities. Furthermore, this study considered subjective social status an indicator of students' social class. While educational research has traditionally regarded parental educational attainment as a primary indicator of social class, the inclusion of a subjective measure of social class may offer a more nuanced understanding of how social, economic, and cultural resources—and their relationship with psychological factors—contribute to students' educational success.

In this study, we hypothesized that social class would be negatively associated with ASC. The results partially supported this hypothesis. Specifically, students who reported lower subjective social status had lower levels of perceived academic competence. However, generational status was not found to be significantly associated with ASC. This may be attributed to the fact that upon entering higher education, students' academic experiences tend to become less influenced by their parents' educational background. FG (first-generation) university students have activated upward-mobility processes, which may positively influence their self-perceptions. This could explain why, in our study, the relationship between being an FG student and ASC (although not statistically significant) was positive. It is then possible that moderators (e.g., AGs) may help explain this relationship. Some previous research had demonstrated that generational status interacts with AGs to account for students' educational outcomes, including ASC (King et al., 2024). However, we found limited research that examined this relationship within academic university contexts. Given this gap, and considering the central role of ASC in the relationship between SES and students' achievement (Chevalère et al., 2023; King et al., 2024; Li et al., 2020; Marsh et al., 2023), this issue was further investigated in Study 2.

Furthermore, this study demonstrated that low subjective social status was indirectly associated with students' educational expectations *through* ASC. However, no direct association was found between low subjective social status and educational expectations, suggesting that perceptions of academic competence play a crucial role in shaping low-SES students' educational experiences. Low social, economic, and cultural resources reduce students' perceptions of being competent in the academic context, which, in turn, contribute to reducing their perceived likelihood of completing university. Conversely, the relationship between SES, ASC, and educational expectations was not significant when students' generational status was considered. In higher education, when FG students are engaged in social mobility processes, their parents' educational background appears to play a marginal role in their educational experiences. These findings suggest that future research into motivated behavior in university settings should place greater emphasis on subjective indicators of social class in order to provide a more nuanced picture of the psychological processes that characterize the experience of students from low socioeconomic backgrounds.

## 5 Study 2

In Study 2 we explored the role of AGs in the association between students' social class and ASC. In fact, Study 1 revealed that subjective social status can be linked to ASC. However, the relationship between students' social class and ASC was non-significant when generational status was taken into account. In this context, the literature indicated that AGs moderate the relationship between students' generational status and educational outcomes (e.g., Bruno et al., 2019; Darnon et al., 2018a,b; King et al., 2024; Smeding et al., 2013). However, to the best of our knowledge, only one study to date has examined the outcome associated with this interaction in relation to ASC (King et al., 2024). Most studies in this area have focused primarily on factors that explain educational inequalities regarding academic achievement (e.g., Bruno et al., 2019; Darnon et al., 2018a,b). Furthermore, the role of subjective social status in its interaction with AGs had not yet been explored in the literature.

Based on studies that had investigated the role of generational status and AGs in students' academic achievements (e.g., Bruno et al., 2019; Darnon et al., 2018a,b; King et al., 2024; Smeding et al., 2013) and considering that academic achievements are strongly related to ASC (Wu et al., 2021), we hypothesized that AGs would moderate the direct association between generational status and ASC. Specifically, no hypotheses were formulated regarding the approach dimensions of AGs. In fact, the role of MAp goals in the outcomes associated with students' generational status remains unclear. On the one hand, some studies suggested that MAp goals favor CG students (e.g., King et al., 2024). On the other hand, some research indicated a slightly stronger relationship between MAp goals and academic achievement for FG students (Darnon et al., 2018a,b). At the conceptual level, some authors have suggested that MAp goals may be particularly helpful for FG students in navigating challenging situations in learning environments (e.g., Darnon et al., 2018a,b). However, other scholars have argued that low-SES students may struggle to fully benefit from MAp goals due to barriers stemming from a lack of social, economic, and cultural resources (e.g., Berger and Archer, 2015; King et al., 2024). Furthermore, no research had examined these relationships while using subjective social status indicators.

Regarding PAp goals, while Darnon et al. (2018a,b) found them beneficial for CG students, other researchers have suggested that the approach dimensions of performance goals may be particularly useful for students from low SES backgrounds who—perceiving limited control over their environment—may rely on external standards to assess their academic performance (e.g., Berger and Archer, 2015). Moreover, regarding PAv goals, in line with Bruno et al.'s (2019) study, we hypothesized that such goals would be detrimental to the ASC of all students, particularly FG students. Focusing on avoiding failure rather than striving for success may intensify feelings of inadequacy and diminish the perception of academic competence for FG students. No hypotheses were formulated concerning MAv goals, as there were no studies that had examined these types of educational goals in relation to students' social class. Finally, in light of the dearth of studies which examined the moderating role of AGs in the relationship between subjective social class and educational outcomes, no hypotheses were formulated.

### 5.1 Method

#### 5.1.1 Participants

We conducted an a priori power analysis to determine the appropriate sample size to achieve a statistical power of 0.80, assuming a small-to-medium effect size and an alpha of 0.05 in a regression model with five predictors (Soper, 2024). The results indicated that a sample size ranging from 91 to 261 participants would suffice for this study.

Two hundred and seventy-six Italian undergraduate psychology students participated in this study. Only 270 students completed all measures (*M*_age_ = 20.26, *SD*_age_ = 1.28; 79% females; four participants did not provide information about their age; three participants did not provide information about their gender). Informed consent was obtained from all participants before starting the online survey. This study was approved by the university ethics committee and adhered to ethical standards for psychological research.

#### 5.1.2 Measures

Students' generational status (CG students: *N* = 138; FG students: *N* = 132), subjective social status, and ASC (ω = 0.82) were measured as in Study 1. AGs were assessed by using an adaptation of Cecalupo et al.'s (2022) scale from high schools to academic university contexts. The measure consists of 12 items, with three items for each dimension: MAp goals (ω = 0.68), PAp goals (ω = 0.89), MAv goals (ω = 0.80), and PAv goals (ω = 0.80). An exploratory factor analysis, conducted using maximum likelihood extraction and oblimin rotation, revealed the presence of four factors with factor loadings ranging from 0.573 to 0.981. Confirmatory factor analysis indicated the good fit of the four-factor model to the data ($\chi_{\left(48\right)}^{2}$ = 141.405, *p* < 0.001, CFI = 0.93, TLI = 0.91, RMSEA = 0.08, SRMR = 0.06).

### 5.2 Data analysis and results

We conducted all analyses with Jamovi software (The Jamovi Project, 2024) and the GAMLj module (General Analyses for Linear Models; Gallucci, 2019). Using four general linear models, we evaluated the associations between social class (generational status and subjective social status), AGs, and their interactions with ASC. Four separate models were tested for each AG.

After preliminary data processing (Tabachnick and Fidell, 2013), statistical descriptives and correlation analyses were carried out (see Table 2).

**Table 2:** Descriptive statistics and correlation analyses.

**Variables**	**M**	**SD**	**1**	**2**	**3**	**4**	**5**	**6**	**7**
1. GS			–						
2. L-SSS	4.627	1.405	0.351^***^						
3. ASC	5.876	1.042	−0.050	−0.281^***^					
4. MAp	4.547	0.505	0.031	0.050	0.114				
5. PAp	2.348	0.960	−0.064	−0.056	0.230^***^	0.004			
6. MAv	3.627	0.905	0.081	0.154^*^	−0.245^***^	0.302^***^	0.083		
7. PAv	2.333	0.996	−0.052	−0.042	0.044	0.044	0.535^***^	0.240^***^	

*** *p* < 0.001.

* *p* < 0.05.

GS, generational status (0 = Continuing- Generation; 1 = First-Generation); L-SSS, low subjective social status; ASC, academic self-concept; Map, mastery-approach goals; Pap, performance-approach goals; MAv, mastery-avoidance goals; PAv, performance-avoidance goals.

As in Study 1, generational status (0 = CG; 1 = FG) was positively correlated with low subjective social status. Low subjective social status was negatively correlated with ASC. Furthermore, low subjective social status was weakly correlated with MAv goals, but no significant associations were found between low subjective social status and the other AGs. Similarly, and in line with previous studies (e.g., Darnon et al., 2018a,b), generational status was not correlated with AGs. ASC was positively correlated with PAp goals and negatively correlated with MAv goals. MAp and PAv goals showed no significant correlations with ASC.

The results of the general linear models appear in Table 3.

**Table 3:** General linear model results.

	**Dependent variable: academic self-concept**					
**Predictors**	**b**	**SE**	**β**	**t**	**p**	**95% CI**
**Model 1**						
GS	0.106	0.130	0.102	0.819	0.413	[−0.149, 0.362]
L-SSS	−0.226	0.046	**−0.304**	−4.862	<0.001	[−0.317, −0.134]
MAp	0.348	0.174	**0.169**	2.004	0.046	[0.006, 0.691]
GS^*^MAp	−0.190	0.252	−0.092	−0.753	0.452	[−0.686, 0.306]
L-SSS^*^MAp	−0.083	0.090	−0.056	−0.918	0.359	[−0.261, 0.095]
**Model 2**						
GS	0.142	0.128	0.136	1.110	0.268	[−0.110, 0.393]
L-SSS	−0.221	0.046	**−0.298**	−4.861	<0.001	[−0.311, −0.132]
PAp	0.222	0.063	**0.204**	3.499	<0.001	[0.097, 0.347]
GS^*^PAp	−0.192	0.137	−0.176	−1.408	0.160	[−0.461, 0.077]
L-SSS^*^PAp	0.011	0.048	0.014	0.223	0.824	[−0.084, 0.106]
**Model 3**						
GS	0.139	0.128	0.133	1.087	0.278	[−0.113, 0.392]
L-SSS	−0.206	0.046	**−0.278**	−4.463	<0.001	[−0.297, −0.115]
MAv	−0.236	0.067	**−0.205**	−3.513	<0.001	[−0.368, −0.104]
GS^*^MAv	−0.104	0.139	−0.090	−0.749	0.455	[−0.378, 0.170]
L-SSS^*^MAv	0.056	0.049	0.069	1.142	0.254	[−0.041, 0.154]
**Model 4**						
GS	0.134	0.130	0.129	1.033	0.303	[−0.122, 0.390]
L-SSS	−0.233	0.046	**−0.313**	−5.014	<0.001	[−0.324, −0.141]
PAv	0.161	0.085	0.154	1.895	0.059	[−0.006, 0.328]
GS^*^PAv	−0.309	0.128	**−0.295**	−2.412	0.017	[−0.561, −0.057]
L-SSS^*^PAv	0.066	0.042	0.089	1.557	0.121	[−0.017, 0.150]
**Simple effects of GS**						
PAv −1SD	0.442	0.185	**0.424**	2.386	0.018	[0.077, 0.807]
PAv +1SD	−0.174	0.179	−0.166	−0.969	0.333	[−0.526, 0.179]

GS, Generational status (0 = Continuing-Generation; 1 = First-Generation); L-SSS, low subjective social status; MAp, mastery-approach goals; PAp, performance-approach goals; MAv, mastery-avoidance goals; PAv, performance-avoidance goals. Significant coefficients are in bold.

In *Model 1*, low subjective social status was negatively associated with ASC. Students' generational status was not associated with ASC. MAp goals showed a small positive association with ASC. The interaction between social class (generational status and subjective social status) and MAp goals was non-significant. The model explained 8.8% of the variance in ASC.

In *Model 2*, low subjective social status was negatively associated with ASC. Students' generational status was not associated with ASC. PAp goals were positively associated with ASC, while the interaction between social class (generational status and subjective social status) and PAp goals was non-significant. The model explained 11.9% of the variance in ASC.

In *Model 3*, low subjective social status was negatively associated with ASC. Students' generational status was not associated with ASC. MAv goals were negatively associated with ASC. The interaction between social class (generational status and subjective social status) and MAv goals was non-significant. The model explained 11.2% of the variance in ASC.

Finally, in *Model 4*, as in the previous models, low subjective social status showed a negative association with ASC, whereas students' generational status was not directly associated with ASC. PAv goals were not associated with ASC. However, an interaction emerged between generational status and PAv goals. Specifically, the association between generational status and ASC was positive only when PAv goals were low. These results suggest that FG students experience an improvement in their perceptions of academic competence when they do not focus on avoiding being worse than others. The model explained 8.8% of the variance in ASC.

### 5.3 Discussion

In this study, we investigated psychological factors that influence the relationship between social class and academic competence perceptions, considering both objective (parents' educational attainment) and subjective (subjective social status) SES indicators. Specifically, the aim of this study was to explore the moderator role of AGs in the relationship between social class and ASC.

Confirming the results of Study 1, low subjective social status was negatively associated with ASC. Students' subjective perceptions of their social standing within society can undermine their sense of academic competence in university contexts, regardless of both parents' educational attainment and AGs. On the contrary, being an FG (first-generation) student was not associated with ASC. It is worth noting that, like in Study 1, the (non-significant) relationship between students' generational status and ASC showed a positive trend, suggesting that these students could experience positive self-perceptions based on the fact that, compared to their parents, they had activated the social elevator and were involved in social mobility processes. However, given the partial nature of this finding, future research should further explore the experience of FG students at university with the aim of shedding light on their life experiences as a source of potential strength—instead of a vulnerability condition—and identify factors that can promote their psychological resilience (e.g., Hernandez et al., 2021).

Regarding the AGs, our results revealed that, net of the associations between social class and students' perceived academic competence, motivational dimensions play a significant role in shaping students' ASC. Among mastery goals, the avoidance dimension was negatively associated with students' perceptions of their academic competence. While MAv goals remain relatively underexplored in the educational literature, this study has highlighted their relevance, particularly as a potential risk factor for ASC. We thus recommend that future research continues to include this dimension when examining motivation-related outcomes, especially within academic contexts. Instead, MAp goals were only weakly positively associated with ASC in this study. Contrary to the crucial role that the approach dimension of mastery goals generally plays in students' adaptation, our study suggests that MAp goals have a limited impact on students' perceptions of academic competence. These findings are consistent with previous studies that highlighted that MAp goals are motivational drivers only weakly related to academic achievements and competencies, in which social-comparison processes are involved (Marsh, 1990, 1993), and more strongly associated with intrinsic motivation and enjoyment of learning (e.g., Bieg et al., 2017; Niepel et al., 2014). Nevertheless, further research is needed to better understand which factors may activate the positive outcomes typically associated with MAp goals, even in contexts where social-comparison processes are salient.

Regarding performance goals, consistent with the previous research (e.g., Niepel et al., 2014), PAp goals were positively associated with students' perceptions of competence. This suggests that the desire to outperform peers at university influences how students perceive themselves within that academic context. These findings align with the conceptualization of ASC (Marsh, 1993; Seaton et al., 2010), which posits that students' academic self-perceptions are grounded in comparative performance evaluations. In contrast to other AGs, PAv goals were not directly associated with ASC. Although PAv goals are generally considered the least-adaptive forms of academic motivation, they appear not to be negatively related to ASC. Compared to the approach dimension of performance goals, PAv goals are probably less relevant for students' self-perceptions of competence, as primarily cognitive constructs, but may play a greater role in explaining emotional states (e.g., Pekrun et al., 2006). Furthermore, in our study, the lack of association between PAv and ASC may depend on the educational level at which the research was conducted. In lower-school cycles, in which PAv appeared to be negatively related to ASC (see, Niepel et al., 2014, for a longitudinal study conducted with secondary school students), learning environments are typically structured around smaller class sizes compared to university settings. In such social contexts, processes of social comparison may be more salient, potentially contributing to the relationship between PAv and ASC.

Overall, this study confirmed the important role that AGs can play in students' self-perceptions (Niepel et al., 2014), even within academic contexts. The fact that AGs were associated with academic-related outcomes even when accounting for different indicators of social class underscored the central role of motivation in students' adaptation across all levels of education.

An additional interesting finding in this study concerns the role of PAv goals in the relationship between social class and ASC. PAv goals moderated the relationship between generational status and ASC. Compared to students whose parents hold a university degree, FG students can report higher ASC when their PAv goals are low: lower motivation to avoid academic failure can act as a promotive factor of their ASC. On the other hand, PAv goals did not play any role in the relationship between subjective social status and ASC. Regardless of the specific dimensions of AGs, in our study, motivational factors appeared unable to modify the negative relationship between belonging to a low social status and students' academic self-perceptions.

In order to explain these results, we formulated some hypotheses that might be tested in future research. A first point to consider is that PAv goals moderated only the relationship between generational status and ASC, whereas they did not play a role in the relationship between subjective social status and ASC. This result may depend on the measures used to assess students' social class and thus on the psychosocial dimensions that these measures are able to capture. Subjective social status reflects a broad and complex perception of the social position an individual occupies in society, whose negative effects are likely difficult to counterbalance by referring only to individual factors—such as motivation. In this study, in fact, regardless of AGs, perceiving oneself as being in a disadvantaged socioeconomic condition appeared to be associated with a negative academic self-perception. Future studies should therefore account for additional contextual moderating variables in the relationship between subjective social status and academic outcomes, such as sources of social support (e.g., friends) or the level of integration achieved within academic university contexts (e.g., undergraduates' academic socialization; Farnese et al., 2022). Moreover, we can hypothesize that students' generational status, as a direct measure of a specific aspect of their family background— namely, the continuity of academic experiences between parents and children—represents, on the one hand, a less-comprehensive indicator of SES compared to subjective social status (which includes both psychological and social dimensions), and, on the other hand, a measure that is more sensitive to variables specifically related to the academic context, such as AGs. In this regard, some studies (Bruno et al., 2019; Darnon et al., 2018a,b; King et al., 2024; Smeding et al., 2013) have shown that AGs interact with objective socioeconomic indicators, such as the parents' level of education. In particular, Bruno et al. (2019) found that negative outcomes associated with PAv goals tended to emerge especially when failure was made salient. In the absence of threats to success, however, PAv goals were not related to students' academic achievements. It is therefore possible that, in our study, the lack of information on students' academic progress or on material or psychological barriers perceived as obstacles to success prevented the emergence of the negative outcomes associated with high levels of PAv goals. In order to clarify these mechanisms, future studies should collect more detailed information on students' university experiences, such as academic performance, consistency in study progression, difficulties in study-related activities, or the degree of integration within the university environment. Finally, regarding the positive role of low levels of PAv goals in the ASC of FG students, it can be hypothesized that for these students—who are often poorly socialized to university environments by parents who, before them, never attended university academic environments—university represents an uncertain path. In light of the negative outcomes associated with PAv goals and the vulnerability of FG students to these AGs under certain conditions (Bruno et al., 2019, 2020), these students may benefit from being the first in their family to attend university, developing positive academic self-perceptions, only when maladaptive AGs (i.e., PAv goals) are low. In view of the significance of these findings, we hope future research further explores the role of the family context in students' academic experiences, especially with regard to socio-psychological dynamics related to PAv goals.

## 6 General discussion

With two cross-sectional studies, in the present research we examined psychological mechanisms that may be involved in the relationship between students' social class and academic adaptation in academic university contexts. Since the literature paid little attention to the role of subjective social status in university students' adaptation (Diemer et al., 2013), we also explored outcomes associated with self-perceived aspects of social class beyond traditional indicators, such as parents' educational attainment. Furthermore, with this research we aimed to address a gap in the literature which had primarily examined the outcomes related to social class in terms of academic achievement. In this regard, international research has recognized the need to explore outcomes more closely tied to perceptions of success, since individuals from low social classes often face barriers to academic and career success regardless of their academic achievement (OECD, 2019, 2023, 2024a). Overall, the present research has contributed to expanding the existing literature on the role that structural factors can play in students' academic pathways, influencing motivational processes and, consequently, their likelihood of academic success.

To accomplish our goals, in Study 1 we aimed to shed light on the relationship between students' social class and university students' perceptions of academic competence. Although prior research had demonstrated that family SES exerts a negative influence on students' performance and self-perceptions during middle and high school, this field of investigation remains little-explored within academic university contexts (Chevalère et al., 2023; King et al., 2024; Li et al., 2020; Marsh et al., 2023). A further relevant aspect of this study was the attention paid to students' educational expectations. In particular, psychological and educational scholars have highlighted that educational expectations play an important role in the academic adaptation of young people. The presence of positive attitudes toward the educational experience represents a fundamental resource for all students. In the presence of high expectations, in fact, the commitment to carrying out a task increases and, consequently, there is a greater probability of overcoming adverse events and barriers of various types (Wigfield and Eccles, 2000). For this reason, in this study we examined the indirect association between students' social class and their educational expectations *through* academic self-concept.

Following the findings of the extant literature (e.g., Chevalère et al., 2023; King et al., 2024; Li et al., 2020; Marsh et al., 2023), our results confirmed that belonging to a low-status social class can be negatively associated with students' perceptions of academic competence, thus limiting their future educational success. Given that no direct association was identified between students' social class and educational expectations, our findings underscore the pivotal role of perceived academic competence in university students' adaptation. What is especially noteworthy is that, in contrast to prior studies on this topic, the present study incorporated both subjective and objective social-class indicators. Despite some past evidence having indicated that the individual's socioeconomic and cultural position within society is a more reliable predictor of wellbeing than objective measures (Tan et al., 2020), these aspects have been largely overlooked in the educational literature (Diemer et al., 2013). The findings of our study indicated that the indirect relationship between social class and students' educational expectations via ASC was significant only when subjective social status was taken into account. Students' adaptation appears to be primarily influenced by their social, economic, and cultural resources rather than the educational background of their parents. Consequently, it can be argued that social, economic, and cultural capital play a pivotal role in facilitating or impeding the adaptation of young people, even once they have entered the academic university context.

In Study 2 we aimed to explore the role of motivational factors, specifically AGs, in the relationship between students' social class and ASC. Considering the crucial influence of ASC on educational outcomes (e.g., Marsh et al., 2023), our primary goal was to assess whether the motivational factors that typically drive students' engagement in academic environments could moderate the link between social class and ASC. Thus, in line with literature that had indicated that AGs may have either positive or negative consequences on students' adaptation, which depend on students' characteristics, particularly their social class (e.g., Bruno et al., 2019; Darnon et al., 2018a,b; King et al., 2024; Smeding et al., 2013), in this study we aimed to assess whether AGs would act as risk or protective factors in academic adaptation.

The findings corroborated those of Study 1, confirming a negative association between subjective social status and ASC as well as the absence of an association between parents' educational attainment and ASC. Furthermore, AGs did not play a significant role in the relationship between subjective social status and ASC. This indicates that the perception of having limited socioeconomic and cultural resources may negatively impact students' self-concepts in academic contexts, regardless of the parents' educational attainments and AGs. All in all, these results suggest that the perception of lacking adequate socioeconomic and cultural resources represents a risk factor for academic adaptation.

A further interesting finding from Study 2 was the significant interaction that emerged between social class and AGs, specifically when our analysis focused on parental educational attainment and only in relation to PAv goals. Specifically, contrary to our hypothesis, the results did not indicate that FG status led to a negative perception of academic competence when PAv goals were high. However, we found that when PAv goals were low—namely, when academic motivation was not driven by the fear of being worse than others—FG students reported higher ASC than their CG peers. This suggests that, without the pressure of fearing failure or appearing inferior compared to their peers, FG students are able to build a positive self-image and feel confident in academic domains. As discussed in Section 5.3, this result can be interpreted by considering that an objective indicator of SES (e.g., parents' educational level) does not in itself represent a barrier to students' academic self-perceptions. When FG students are less concerned with avoiding competition to outperform others, they may even benefit from being the first in their family to reach tertiary education, which can become a source of pride and personal achievement. However, further studies are needed to better understand the role of PAv goals in tertiary education pathways, taking into account students' broader social contexts. As other studies have also suggested, students' generational status may influence their PAv goals (Jury et al., 2015). It can be hypothesized that this relationship is further mediated by multiple factors related to the student's familial environment, such as parental involvement (Kim et al., 2020; Kim, 2022; Wilder, 2014). In this regard, the literature has shown that structural factors play a role in shaping parents' attitudes, goals, and behaviors toward their children's educational experience (e.g., Cecalupo et al., 2024; Kim et al., 2020). These suggestions are, however, speculative in nature, and further studies are needed on the role of families in relation to students' self-perceptions in university contexts.

Overall, the findings of this research highlight that subjective social status can hinder students' adaptation, even once they reach academic university contexts. Perceived socioeconomic and cultural resources emerged as a more important dimension than generational status in understanding students' experiences during their university studies. It is likely that by the time they enter higher education, these students have already overcome the educational barriers posed by their family's educational background, rendering their perceived socioeconomic and cultural resources more significant for academic adaptation. Nevertheless, although some research had shown that parents' educational attainment can hinder university students' adaptation, low maladaptive AGs—which are oriented toward social comparison and the avoidance of being worse than others (i.e., PAv goals)—can facilitate adaptive processes. This suggests that, in certain circumstances, FG students may demonstrate greater levels of adaptation than their counterparts, despite the detrimental impact of low SES on their academic self-perceptions. When the processes of social mobility are initiated—namely, when FG students attain tertiary education—not focusing on the goal of not performing worse than others can enable these students to better express themselves on their educational pathways. In this regard, also considering that in both studies of this research parents' educational attainment showed a positive association (although not statistically significant) with students' ASC, future research should continue to explore psychological mechanisms that may explain the role of social class in academic adaptation by adopting a perspective focused on how the shared life experiences of students from lower social-status backgrounds can serve as a source of resilience (e.g., Hernandez et al., 2021).

In conclusion, this research has underscored the importance of recognizing the role of SES in shaping university students' adaptation and academic success. The findings also imply a need to analyze the underlying processes (e.g., ASC and AGs) that influence the academic experiences of low-SES students. Further research in this direction is needed to better understand the socio-psychological mechanisms that facilitate or hinder the adaptation of students from disadvantaged socioeconomic and cultural backgrounds. In this regard, more attention should be paid to variables related to the subjective perception of one's socioeconomic condition, which appear to have a more direct and significant impact on students' wellbeing compared to ascriptive variables (i.e., parental education level). Understanding why and how subjective social status influences students' university experiences can provide valuable insights for developing targeted interventions aimed at mitigating its negative effects. At the same time, it is important not to overlook more objective and ascriptive social-class variables and to identify the specific contexts and circumstances in which they exert a stronger influence on students' adaptation and academic achievement. An integrated understanding of both subjective and objective dimensions of SES in shaping students' academic success can offer a more comprehensive perspective on factors that affect students' success within academic university environments.

## 7 Practical implications

The findings of this research offer valuable insights for informing educational policies and practices at the tertiary education level. Overall, this research revealed that the perception of few socioeconomic and cultural resources was negatively associated with students' academic adaptation. This suggests that even when students have initiated social-mobility processes by enrolling in university, their education paths may be jeopardized by a lack of resources. Consequently, at the political level, it is essential to reinforce social policies that address the specific social, economic, and cultural needs of students from low socioeconomic backgrounds and their families. Additionally, university communities can support the adaptation of students from low socioeconomic backgrounds by acting on three levels: students, teachers, and learning environments.

In the first place, universities should provide support services tailored to the needs of students from low socioeconomic backgrounds. For example, counseling services, provided throughout students' entire academic careers, may play a crucial role in supporting their academic adaptation. As research findings have indicated that socioeconomic and cultural barriers hinder students' adaptation primarily due to their association with underlying psychological barriers (e.g., Duffy et al., 2016; Marini et al., 2023), psychological support may be particularly beneficial for students' resilience. Thus, counseling services should incorporate interventions aimed at increasing students' awareness of how social class influences their academic experiences, while also supporting them in identifying the economic, social, and cultural resources they can draw upon to navigate university successfully (e.g., Stephens et al., 2014, 2017). Such interventions, fostering students' psychological resources, may enhance their perceptions of academic competence by emphasizing that academic performance is not determined solely by individual traits; rather, structural factors—such as social status or country of origin—can negatively impact students' academic achievement.

Furthermore, teachers, as “agents of social change” (Butera et al., 2021), can actively shape the academic experiences of students from impoverished socioeconomic backgrounds not only by being mentors and supporting them at socioemotional levels, but also by facilitating the students' integration into university contexts (Farnese et al., 2022; Tinto, 2022). For instance, the adoption of cooperative teaching methodologies may promote knowledge sharing, strengthen social relationships, and foster the development of peer networks among all students (Mendo-Lázaro et al., 2022). These teaching-learning strategies may be particularly suitable for students from low socioeconomic backgrounds who are generally socialized by their families to appreciate the values of interdependence (Stephens et al., 2012). In these contexts, students from low socioeconomic backgrounds may benefit from peer support to develop a positive self-concept and overcome the barriers that often characterize their life experiences. Consequently, in light of the crucial role that teachers play in students' academic adaptation, academic institutions should provide tailored training to support them effectively. For example, teachers should be offered professional development programs that address the relationship between social class and academic performance, with the aim of helping teachers gain a deeper understanding of how their beliefs, attitudes, and behaviors may influence the educational trajectories of students from low socioeconomic backgrounds (see the second-order effects in the Social Class Academic Contexts Mismatch Model in Goudeau et al., 2025).

Finally, academic communities can facilitate the adaptation process of students from low-resourced socioeconomic backgrounds by enhancing the quality of learning environments, with the goal of discouraging maladaptive forms of academic motivation. Research within the framework of achievement goal theory (Urdan and Kaplan, 2020) suggests that the achievement gap based on SES may be at least partly attributed to how university systems evaluate students' performance (e.g., Autin et al., 2019; Butera, 2006; Smeding et al., 2013). In fulfilling their selective function (Autin et al., 2015, 2019), in fact, grades can trigger social-comparison processes, motivating students to engage in academic activities with the goal of either striving to outperform their peers (PAp goals) or avoiding underperformance (PAv goals) (e.g., Cecalupo et al., 2022; Marsh et al., 2017; Pulfrey et al., 2011; Smeding et al., 2013; Stephens et al., 2017). For this reason, achievement goal theory scholars emphasize the importance of modifying learning environments (Ames, 1992; Ames and Archer, 1988), reinforcing the culture of formative assessment. For instance, linking grades to clear feedback on students' individual progress—highlighting both strengths and areas for improvement—may reduce students' focus on performance-based AGs and foster motivation more conducive to learning (Butera et al., 2024; Cauley and McMillan, 2010; Morris et al., 2021; Smeding et al., 2013). Such learning environments may benefit all students, especially those with difficult socioeconomic backgrounds. In support of this, the present research (Study 2) showed that when AGs focused on avoiding being worse than others (PAv goals) were low, FG students perceived themselves as more competent than their CG peers.

In conclusion, this research suggests that interventions aimed at facilitating the academic adaptation of students from challenging socioeconomic backgrounds should engage both the educational community and the institutional context. The shared goal should be to reduce, and ideally eliminate, the negative consequences associated with the lack of socioeconomic and cultural resources by strengthening the psychological resources of socioeconomically- and culturally-disadvantaged students and providing them with the emotional and instrumental support necessary for successfully navigating their academic experiences.

## 8 Limitations and future research

This research had several limitations that should be taken into account when interpreting the presented results. First, the cross-sectional nature of this research limited our ability to draw conclusions about the direction of the relationships between the variables examined. This is an important aspect to explore in future research, particularly through more complex designs (e.g., experimental and longitudinal studies). For example, complex methodological approaches would be useful for exploring in-depth the relationship between AGs and academic self-concept, which may be bidirectional. In addition, longitudinal studies could help to examine how the psychological processes explored in this research may develop or remain stable over time. In this context, conducting studies that span the entire duration of students' academic careers would be valuable for gaining a more comprehensive understanding of potential sensitive periods of student adaptation. Longitudinal research would also allow scholars to identify additional factors that influence the adaptation of students from low SES backgrounds over time. One might hypothesize, for example, that “effective” academic socialization (Farnese et al., 2022; Tinto, 2022) may play a key role in facilitating the academic integration of students from low-SES families. Moreover, the development of positive relationships with peers could discourage social-comparison processes and foster adaptive forms of motivation. Additionally, a supportive and collaborative university climate could serve as a valuable source of social support for all students, especially those from disadvantaged socioeconomic backgrounds. In sum, tracking the academic paths of these students over time could provide valuable insights into the mechanisms and processes that can hinder or, conversely, facilitate these students' academic success.

Second, although this research highlighted the importance of considering SES's both objective and subjective indicators in studying students' adaptation, we accounted for only one dimension of objective SES, namely generational status, neglecting other factors like the parents' occupations and total household income. This limitation may explain why, in both of our studies, generational status had limited explanatory power. It is plausible to hypothesize that, while generational status is commonly used to assess the role of structural factors in shaping students' educational experiences, the parents' educational attainment primarily reflects family-level cultural resources and does not fully capture broader SES. Future studies should incorporate more comprehensive indicators of socioeconomic and cultural status, including both objective measures (such as those used in PISA; Avvisati and Wuyts, 2024) and subjective indicators to provide a more nuanced understanding of the impact of SES on academic outcomes.

Third, although this research addressed several key factors associated with academic adaptation (i.e., SES, ASC, AGs, and educational expectations), we overlooked the impact of social and contextual factors on students' experiences. As noted earlier, relationships with significant others (Bukowski et al., 2020; Marsh et al., 2017), the academic environment (Murayama and Elliot, 2009; Sommet et al., 2015), and institutional factors (e.g., Farnese et al., 2022) play a crucial role in shaping students' educational trajectories, particularly for those from low social-status families. Future studies should examine the role of social and institutional factors alongside psychological dimensions in order to gain a deeper understanding of the experiences of FG and socioeconomically- and culturally-disadvantaged students. Such research would provide a more nuanced perspective on challenges these students encounter in both their personal and academic lives (see, e.g., Duffy et al., 2020; Garriott et al., 2020), ideally facilitating the identification of efficacious strategies to support students in their educational pathways.

Finally, the present research was conducted within a specific cultural context (Italy). Consequently, and also given the limited number of similar studies in other cultural contexts, interpreting our results while assuming a cross-cultural perspective is constrained. Additionally, inconsistencies in the literature regarding the relationships among the variables examined (objective SES, subjective SES, ASC, AGs, and educational expectations) make it difficult to hypothesize the role of cultural factors in our findings, representing another limitation of the present research. Future research should pay more attention to the role of cultural factors in motivated behavior (see Eccles and Wigfield, 2020; Guay, 2016; King et al., 2017, 2018). Nonetheless, it is worth noting that recent study results suggest that, although culture plays a significant role in motivation, the outcomes associated with motivation appear to be universal (see Guay, 2016). Future research could therefore be designed with the aim of understanding, on the one hand, whether the pattern of results that emerged in the present research is also found in other cultural contexts; and, on the other hand, whether and how cultural dimensions influence students' ASC, AGs, and educational expectations, as well as investigating the relationships between these variables and SES.

Notwithstanding the aforementioned limitations, this research has provided valuable insights into the psychological mechanisms associated with social class in academic university settings. Research in this area should continue through the implementation of more complex research designs and with more representative populations, with the goal of providing more generalizable results with which to build best practices to help low-SES students thrive in academic university settings.

## Data Availability

The raw data supporting the conclusions of this article will be made available by the authors, without undue reservation.
